# Extracellular Adenosine Formation by Ecto-5’-Nucleotidase (CD73) Is No Essential Trigger for Early Phase Ischemic Preconditioning

**DOI:** 10.1371/journal.pone.0135086

**Published:** 2015-08-11

**Authors:** Georg Wolff, Richard Truse, Ulrich Decking

**Affiliations:** Department of Cardiovascular Physiology, Heinrich-Heine-University Düsseldorf, Düsseldorf, Germany; Faculty of Medicine &amp; Health Sciences, UNITED ARAB EMIRATES

## Abstract

**Background:**

Adenosine is a powerful trigger for ischemic preconditioning (IPC). Myocardial ischemia induces intracellular and extracellular ATP degradation to adenosine, which then activates adenosine receptors and elicits cardioprotection. Conventionally extracellular adenosine formation by ecto-5’-nucleotidase (CD73) during ischemia was thought to be negligible compared to the massive intracellular production, but controversial reports in the past demand further evaluation. In this study we evaluated the relevance of ecto-5’-nucleotidase (CD73) for infarct size reduction by ischemic preconditioning in *in vitro* and *in vivo* mouse models of myocardial infarction, comparing CD73^-/-^ and wild type (WT) mice.

**Methods and Results:**

3x5 minutes of IPC induced equal cardioprotection in isolated saline perfused hearts of wild type (WT) and CD73^-/-^ mice, reducing control infarct sizes after 20 minutes of ischemia and 90 minutes of reperfusion from 46 ± 6.3% (WT) and 56.1 ± 7.6% (CD73^-/-^) to 26.8 ± 4.7% (WT) and 25.6 ± 4.7% (CD73^-/-^). Coronary venous adenosine levels measured after IPC stimuli by high-pressure liquid chromatography showed no differences between WT and CD73^-/-^ hearts. Pharmacological preconditioning of WT hearts with adenosine, given at the measured venous concentration, was evenly cardioprotective as conventional IPC. *In vivo*, 4x5 minutes of IPC reduced control infarct sizes of 45.3 ± 8.9% (WT) and 40.5 ± 8% (CD73^-/-^) to 26.3 ± 8% (WT) and 22.6 ± 6.6% (CD73^-/-^) respectively, eliciting again equal cardioprotection. The extent of IPC-induced cardioprotection in male and female mice was identical.

**Conclusion:**

The infarct size limiting effects of IPC in the mouse heart *in vitro* and *in vivo* are not significantly affected by genetic inactivation of CD73. The ecto-5’-nucleotidase derived extracellular formation of adenosine does not contribute substantially to adenosine’s well known cardioprotective effect in early phase ischemic preconditioning.

## Introduction

Cardioprotective mechanisms have become a field of intensive research since the first discovery of ischemic preconditioning (IPC) by Murry et al. [1]: Short repetitive periods of ischemia and reperfusion (I/R) before the onset of an infarct-inducing index ischemia were found to greatly alleviate the resulting infarct size in dogs. Several related mechanisms such as ischemic postconditioning [2] and remote ischemic preconditioning [3,4] have been discovered thereafter, but IPC is known to elicit the strongest cardioprotection [5] and could be triggered in all species tested, including humans [6]. Endogenous substances like adenosine [7], opioids [8] and bradykinin [9] are released during myocardial ischemia and have been identified to trigger cardioprotection, if an above-threshold stimulus is applied during repetitive I/R [10,11].

While the adenosine-dependent activation pathway of IPC has mostly been elucidated [12] and adenosine receptors are known to be crucial for IPC induction [13–15], the main site of receptor-relevant adenosine formation during an ischemic event still remains unclear. A massively increased adenosine release from the intracellular space during hypoxia/ischemia due to a rapid adenosine triphosphate (ATP) breakdown was established [16,17]. In addition, a cascade of highly active ecto-enzymes catalyzes extracellular nucleotide breakdown, in which ecto-5’-nucleotidase (CD73) is the rate-determining step from adenosine monophosphate (AMP) to adenosine [18,19]. Extracellular nucleotides originate preferentially from endothelium [20], but also from cells of the blood compartment, including red blood cells [21,22] and leucocytes [23,24]. CD73-derived adenosine contributes to regulation of endothelial barrier function [25–28], immune responses [29–31] and renal function [32–34] among many others [35].

Several studies on the relevance of CD73-derived adenosine in IPC have yielded controversial results using *in vivo* models of enzyme inhibition or genetic disruption of CD73: Some are indicating a major role for the extracellular formation by CD73 in IPC in mice [36,37] with a complete loss of the cardioprotective effect in absence of the extracellular CD73 activity, while others could not confirm this finding [38]. In view of a massively increased ischemic intracellular adenosine production and the simultaneous release of other trigger substances of IPC (opioids, bradykinin), it appeared difficult to understand, how CD73 could be of utmost importance for ischemic preconditioning, and we thus aimed in this study to further elucidate its role. This is—to our knowledge—the first murine study using both *in vitro* and *in vivo* models of IPC and myocardial infarction to evaluate the cardioprotection by IPC in CD73^-/-^ mice in comparison to the wild type (WT). We hypothesized that extracellular adenosine formation by CD73 is not necessary to reach the threshold for cardioprotection by early phase ischemic preconditioning.

## Methods

### Animals

All experiments were performed in accordance with the German guidelines for the use of living animals and were approved by the Landesamt für Natur-, Umwelt- und Verbraucherschutz (LANUV) of Nordrhein-Westfalen, Germany (ref. no. 8.87–50.10.34.08). Male and female C57Bl/6 and CD73^-/-^ mice, 8–12 weeks of age and weighing 20–30 g, were bred at the Tierversuchsanlage of the Heinrich-Heine-University (Düsseldorf, Germany). CD73^-/-^ mice used in the experiments were created in our own laboratory and have been previously described in detail [29].

### Isolated saline perfused hearts

C57Bl/6 mice (female WT and CD73^-/-^) were randomly assigned to IPC- and control groups, the animals were sacrificed and the hearts were rapidly excised and mounted in a Langendorff unit (isolated heart, size 1, Hugo Sachs Elektronik). They were perfused with a 37°C Krebs-Henseleit buffer at a constant pressure of 80 mmHg and paced to a constant heart rate of 600/min. A balloon catheter for measuring left ventricular function was inserted through the mitral valve into the left ventricle and connected to a pressure transducer. All functional data were assessed using a PowerLab and Chart 5 (ADInstruments).

**Fig 1:**
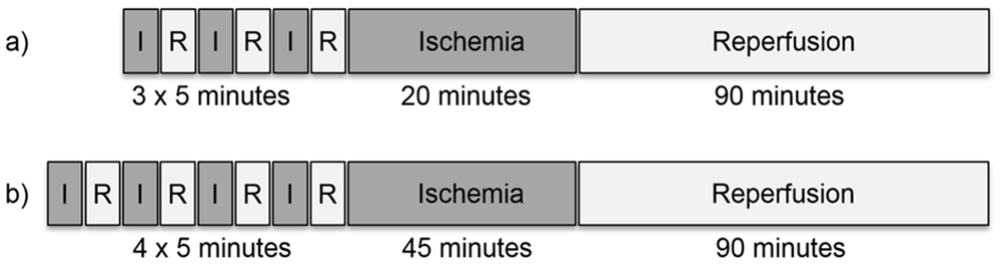
Experimental protocols. a) Experimental protocols for isolated saline perfused hearts and b) *in vivo* experiments.

After having reached equilibrium the hearts were subjected to 3 x 5 min of global ischemia/reperfusion for the induction of IPC (intervention groups only), followed by 20 min of global index ischemia and 90 min of reperfusion (Fig 1). During ischemia the hearts were immersed in 37°C perfusion buffer to prevent cooling while the perfusion was stopped. Coronary venous effluent was sampled immediately after ischemic preconditioning episodes and subsequently analyzed by high pressure liquid chromatography (HPLC). Eventually the hearts were taken off the Langendorff unit and cut into slices for staining and assessment of infarct size.

In another set of experiments, we used an intracoronary infusion of adenosine (2.5 μM in Krebs-Henseleit buffer) to induce pharmacological preconditioning, corresponding to maximal IPC-induced coronary venous effluent adenosine levels.

### *In vivo* open-chest model

We adopted an *in vivo* open-chest model of myocardial infarction first described by Michael et al. [39] and used a hanging-weight system for coronary occlusion [40].

C57Bl/6 mice (male and female WT and CD73—^/-^) were anesthetized with 100 mg/kg body weight Pentobarbital-Na^+^, intubated with a 20G blunt cannula and mechanically ventilated using a Harvard Apparatus MiniVent 845 with a breathing rate of 150/min and a weight-adapted tidal volume of 250–300 μl. The mice were placed in supine position on a heating pad to assure a steady body temperature of 37°C, which was confirmed by a rectal probe coupled to a digital thermometer. Electrodes were adapted to the limbs to allow a constant record of the ECG.

All surgical procedures were performed under a microscope at 3-10x magnification. After anterolateral thoracotomy and identification of the left anterior descending coronary artery (LAD) an 8–0 prolene suture was passed beneath the vessel and threaded through a short piece of PE-10 tube, eventually both ends were attached to a weight of 1 g each. By letting the weights hang freely and thus tightening the suture ischemia could be induced reliably and was verified by paleness of the distal myocardium and immediate ST-elevation in the ECG. Four cycles of 5 min ischemia and 5 min reperfusion to induce IPC were exerted (IPC-groups only) before an index ischemia of 45 min (Fig 1). After 90 min of reperfusion the animals were sacrificed and the hearts were excised and cut into slices for staining and assessment of infarct size.

### Assessment of infarct size

*In vivo* hearts were excised and perfused with buffered saline to wash out remaining blood, then the suture was tightened again and the heart was perfused with an Evans blue staining solution (EB, 2%) to delineate the area at risk (AAR).

Langendorff hearts and *in vivo* hearts were cut into five slices of 1 mm thickness using parallel razer blades guided in a specially made polyethylene block. The slices were washed and immersed in a triphenyltetrazoliumchloride solution (TTC, 1%) at 37°C to stain vital tissue. Then they were mounted between two glass slides spaced 0.8 mm apart to assure constant thickness in all slices and were photographed from both sides.

Measurement of infarct sizes was performed using Image J (Wayne Rasband, NIH) and Adobe Photoshop. Infarcted tissue is presented as % of all ventricular myocardium (corresponding to AAR in Langendorff experiments) or % of myocardium in the AAR as delineated by Evans blue staining (*in vivo* experiments).

### Measurement of adenosine and inosine levels by HPLC

Effluent samples from Langendorff hearts after ischemic preconditioning episodes were collected on ice, weighed and immediately stored at -20°C until further analysis. They were concentrated using solid phase extraction (Sep-Pak C18 columns (Waters)), excess fluid was vaporized and samples were resuspended in destilled water. Nucleoside levels were then analysed in a high pressure liquid chromatography system (HPLC; Waters 600) on a 3.9 x 300 mm column (60 Ångström, Nova—Pak C18, Waters) using an UV Multiwavelength Detector 490e (Waters) at 254 nm.

### Statistical analysis

All data are presented as mean values ± standard deviation; inter-group differences were tested using an analysis of variance (ANOVA) with a post-hoc Bonferroni test for multiple comparisons, or—where appropriate—an unpaired two-sided Student’s t-test. A paired two-sided Student’s t-test was used to analyze within-group changes of cardiac function before/after index ischemia. A two-sided p-value of 0.05 or less was assumed to indicate a significant difference. Data analysis was performed using SPSS and Sigmaplot 11.

## Results

In this study we evaluated the relevance of ecto-5’-nucleotidase (CD73) for infarct size reduction by ischemic preconditioning in *in vitro* and *in vivo* mouse models of myocardial infarction, comparing CD73^-/-^ and wild type (WT) mice.

### Isolated saline perfused hearts

#### Infarct sizes *in vitr*o

**Fig 2:**
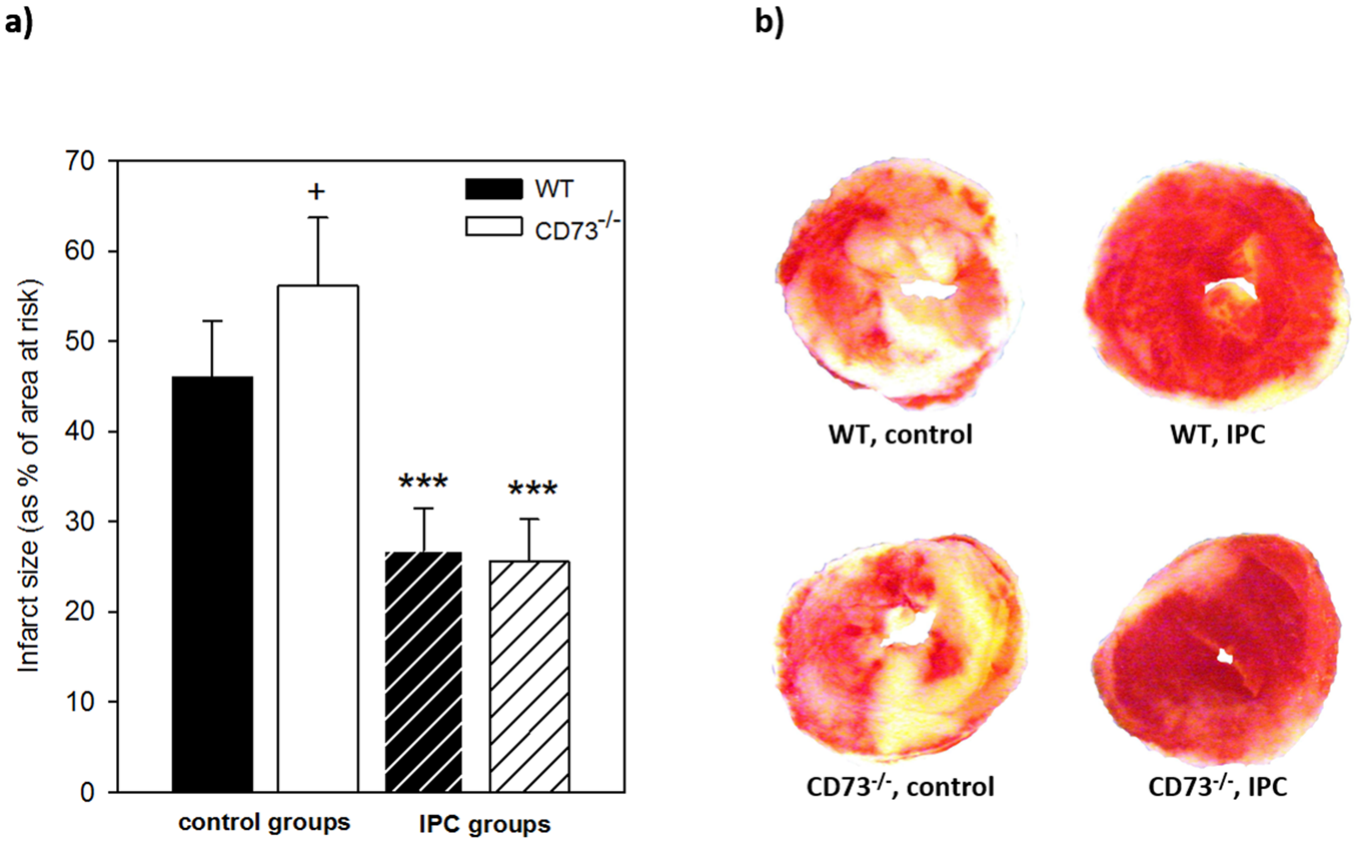
Infarct sizes *in vitro*. a) Infarct size of isolated saline perfused hearts (as % of area at risk, here the whole ventricles) in control groups of wild type (black bars) and CD73^-/-^ mice (white bars) after 20 minutes of index ischemia in comparison to hearts treated with 3x5 minutes of global ischemic preconditioning (IPC, hatched bars) before the onset of the index ischemia. Control infarct size in CD73^-/-^ was bigger than in WT (+, p = 0.02). There was a significant reduction of infarct size by IPC in WT and CD73-deficient hearts compared to the respective control groups (*** = p<0.001). n = 7–8 in all groups. b) TTC staining of transversal heart sections, differentiating infarcted tissue (pale) from vital myocardium (red). Exemplary heart slices of WT and CD73^-/-^ hearts show smaller infarct areas in IPC groups compared to controls.

Isolated saline perfused hearts of WT and CD73^-/-^ mice were exposed to 20 minutes of global index ischemia with or without prior 3x5 minutes of ischemic preconditioning. We found average control infarct sizes of 46 ± 6.3% of all ventricular myocardium in WT and 56.1 ± 7.6% in CD73^-/-^ respectively (n = 7–8 in each group). The infarct size without preconditioning in the CD73^-/-^ group was greater than in WT hearts (p = 0.02). Ischemic preconditioning by 3x5 minutes of repetitive ischemia/reperfusion was able to reduce infarct size to 26.8 ± 4.7% (WT) and 25.6 ± 4.7% (CD73^-/-^), thus eliciting a substantial cardioprotection in both WT and CD73^-/-^ hearts with an average infarct size reduction by 42% (WT) and 54% (CD73^-/-^) in comparison to the respective control groups (n = 7–8, p < 0.001, Fig 2).

#### Left ventricular function

Left ventricular function was assessed with a balloon catheter inserted through the mitral valve in isolated saline perfused hearts (Table 1). There were no significant differences between WT and CD73^-/-^ groups at baseline. Left ventricular end diastolic pressure (LVEDP) and left ventricular developed pressure (LVDP) were significantly increased/decreased (resp.) after index ischemia in WT and CD73^-/-^ hearts of all groups (within-group p<0.01), but we only observed trends to preserved cardiac function by ischemic preconditioning.

**Table 1:** Functional parameters of isolated perfused hearts.

	WT		CD73^-/-^	
	control	IPC	control	IPC
**Coronary flow, baseline (ml/min/g)**	22.7 ± 3.9	19.4 ± 4	17.3 ± 4	18.7 ± 3.6
**Heart weight (mg)**	125.7 ± 16	147.1 ± 35	152.9 ± 20	165 ± 19*
**LVEDP (mmHg), baseline**	10.8 ± 3.2	10.8 ± 4.9	12.2 ± 9.3	11.6 ± 3.4
**LVEDP (mmHg), reperfusion**	36.7 ± 13	29.6 ± 12	48.1 ± 13	35.4 ± 8.2
**LVDP (mmHg), baseline**	108.6 ± 21	117.6 ± 20	120.1 ± 24	122.3 ± 12
**LVDP (mmHg), reperfusion**	41.5 ± 16	56.8 ± 6.3	27.7 ± 14	50.8 ± 15

Coronary flow, heart weight and parameters of cardiac function of WT and CD73^-/-^ hearts in the Langendorff model. Heart weight was higher in CD73-deficient animals of the IPC group compared to wild type controls. Index ischemia significantly worsened cardiac functional parameters within-groups (paired Student’s t-Test: all p<0.01, n = 7–8). There were no significant differences in LVDP, LVEDP and coronary flow between WT and CD73^-/-^ hearts. At reperfusion, preconditioned hearts exhibited only a trend to better cardiac function than corresponding controls, as shown by slight improvement of LVEDP and LVDP. (1-way ANOVA with Bonferroni post-hoc test between all groups, n = 7–8 per group)

* = p<0.05 vs. WT control.

More parameters are shown in Tables in S1 File.

#### Coronary venous adenosine and inosine levels

**Fig 3:**
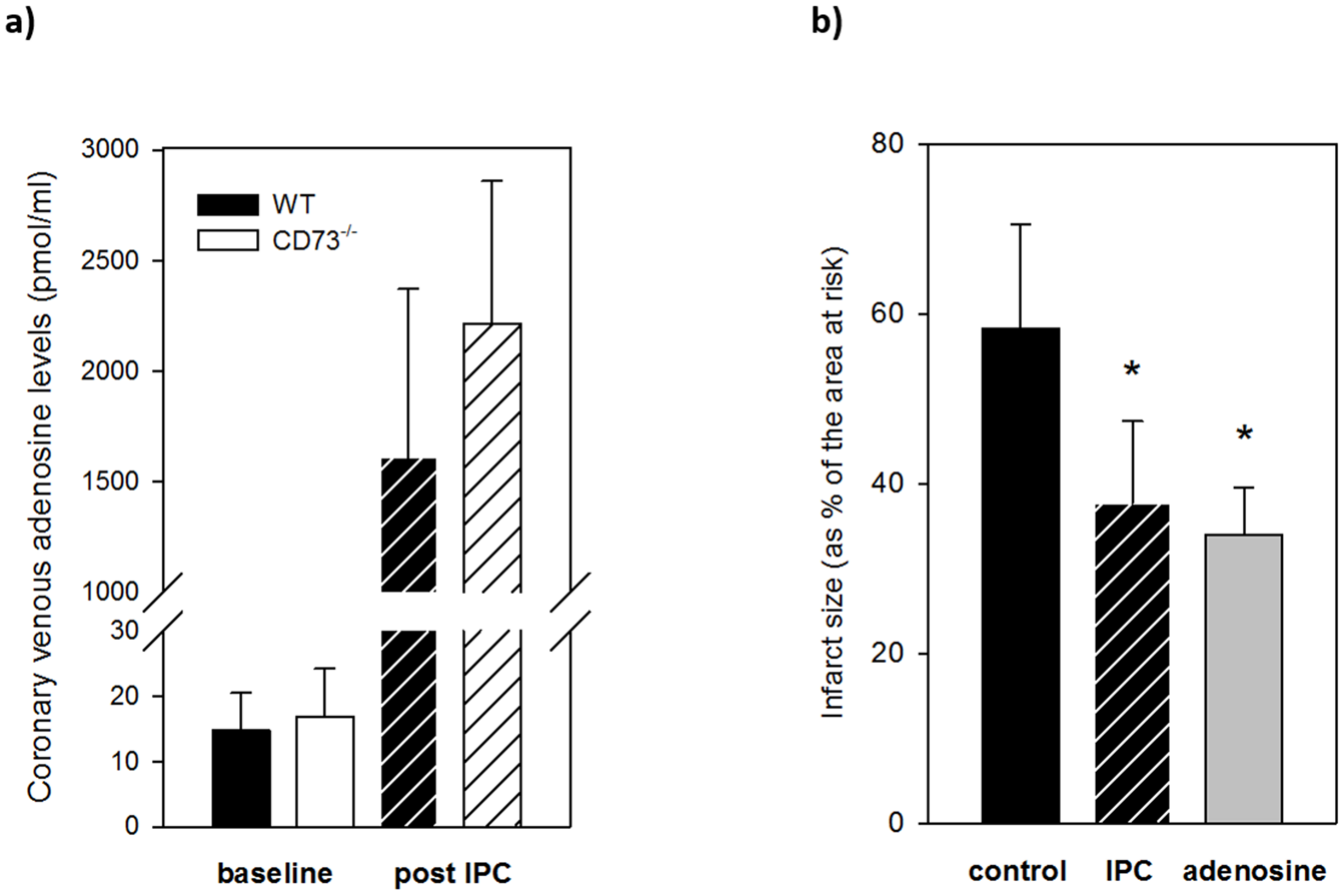
Coronary venous adenosine concentrations and pharmacological preconditioning *in vitro*. a) Coronary venous adenosine concentrations, baseline and maximum immediately post IPC. There was no significant difference between maximum concentrations in WT (black) and CD73^-/-^ hearts (white), with even a trend towards higher maximum adenosine outflow in CD73^-/-^ hearts. n = 7–8 each. b) Infarct sizes (as % of the area at risk, here the ventricular myocardium) in isolated saline perfused wild type hearts after pharmacological preconditioning with an intracoronary infusion of 2.5 μM adenosine (grey bar) for 3x5 minutes in comparison to ischemic preconditioning (IPC, hatched bar) and a control group (black bar). Control n = 8, IPC n = 5, adenosine n = 6, * = p<0.05 compared to the control group.

HPLC analysis of coronary venous effluent was performed to determine nucleoside outflow during IPC (Fig 3a). Contrary to the presumed reduction in CD73^-/-^ hearts due to the lack of extracellular adenosine formation, we found equal basal and maximal levels of effluent adenosine in WT and CD73^-/-^ hearts after IPC stimuli. If anything, there was a trend to slightly higher concentrations in effluent from hearts with genetic deficiency of the enzyme: Basal adenosine concentrations of 14.7 ± 5.8 pmol/ml (WT) and 16.8 ± 7.4 pmol/ml (CD73^-/-^) dramatically increased during IPC, amounting to 1611 ± 759 pmol/ml in WT and 2214 ± 644 pmol/ml in CD73^-/-^ hearts immediately after the first IPC cycle (Fig 3a, n = 7–8 each). Effluent inosine levels increased from 51.6 ± 29 pmol/ml (WT) and 70.1 ± 25.5 pmol/ml (CD73^-/-^) at baseline to 5390 ± 2095 pmol/ml (WT) and 11604 ± 3962 pmol/ml (CD73^-/-^) after IPC (p<0.01, n = 7–8 each), which demonstrates an even higher nucleoside flux in CD73-deficient hearts after short-term ischemia compared to WT.

#### Pharmacological preconditioning by intracoronary adenosine infusion

In order to understand whether the measured coronary venous adenosine concentration could trigger IPC in our setting of the isolated saline perfused heart, we performed another set of experiments using a coronary infusion with a final concentration of 2.5 μM adenosine—corresponding to the maximum measured adenosine concentration during conventional IPC—as a preconditioning stimulus *instead of* short repetitive episodes of I/R. This was compared to a control group and a group treated with conventional IPC in wild type hearts. Pharmacological preconditioning with adenosine resulted in a significant reduction of infarct size after index ischemia to 34 ± 5.5**%** of all ventricular myocardium in comparison to the control group (58.3 ± 12.2% infarct size of ventricular myocardium, p < 0.001), which meant an infarct size reduction by 41.7%, equal to the effects of conventional IPC (37.6 ± 9.7% of all myocardium, infarct size reduction by 35.5%, p < 0.01) (Fig 3b).

### *In vivo* open-chest model

#### Infarct sizes *in vivo*

**Fig 4:**
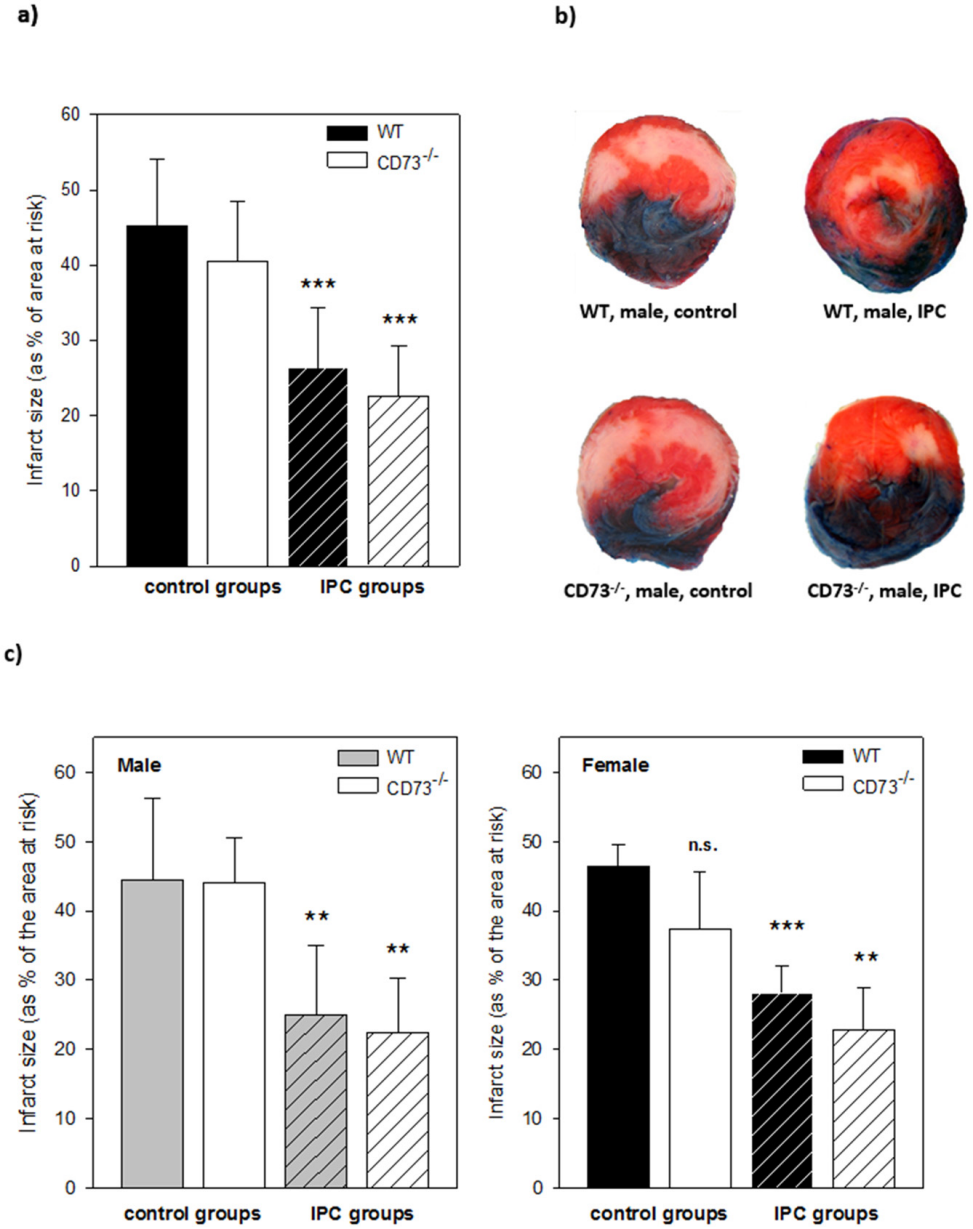
Infarct sizes *in vivo* open-chest model and sex differences. a) Infarct sizes *in vivo* in wild type and CD73^-/-^ mice. Ischemic preconditioning reduced infarct sizes in both IPC groups significantly compared to control groups. n = 12–13 per group, *** = p<0.001 in comparison to the respective control group. b) Double staining of *in vivo* heart slices with Evans Blue (blue dye) and TTC (red dye) to differentiate between infarcted tissue (white), vital cardiomyocytes (red) and tissue outside of the AAR (blue). Exemplary heart slices of male wild type and CD73^-/-^ animals show less infarcted tissue in IPC groups compared to controls. c) Infarct sizes of wild type and CD73^-/-^ mice differentiated by sex: There were no significant differences between male and female mice concerning either control infarct sizes or cardioprotection by IPC. n = 5–7 per group, ** = p<0.01, *** = p<0.001 in comparison to the respective control group.

We established an *in vivo* model of ischemic preconditioning and myocardial infarction to confirm the isolated heart findings in the intact animal. Using the protocol described (Fig 1) we found infarct sizes of 45.3 ± 8.9% of the AAR in the wild type control group, ischemic preconditioning elicited a significant cardioprotection and reduced infarct size to 26.3 ± 8% of the AAR, amounting to a reduction by 41.8% (n = 12, p < 0.001). This was comparable to the data in isolated hearts (Fig 4a). CD73-deficient animals showed no differences to the wild type in control infarct size with an average of 40.5 ± 8% of the AAR. Infarct size was again significantly alleviated to 22.6 ± 6.6% of the AAR in the IPC group (reduction by 44.2%, n = 13, p < 0.001).

#### Sex differences

To exclude possible effects of sex on infarct size and preconditioning effects, male as well as female animals were analyzed *in vivo* (n = 5–7). Male and female wild type and CD73^-/-^ mice showed no significant differences in infarct size in control and IPC groups, ischemic preconditioning was evenly cardioprotective in both sexes (Fig 4c, p < 0.01). A trend to smaller control infarct sizes was observed in female CD73^-/-^ control animals, which did not reach statistical significance.

Raw data and additional parameters can be found in Tables in S2 File.

## Discussion

The aim of this study was the evaluation of extracellular adenosine formation by ecto-5’-nucleotidase (CD73) and its relevance for cardioprotection by early phase ischemic preconditioning, which has been a controversial subject due to conflicting prior reports [36–38]. We made use of two different murine models of IPC and myocardial infarction to assess this issue, and we present the following major findings: in our setting (1) IPC elicits the same infarct size reduction in wild type and CD73 deficient mice both *in vitro* and *in vivo*, (2) after IPC, cardiac coronary venous adenosine outflow is similar in wild type and CD73^-/-^ hearts, inosine outflow is even higher in CD73^-/-^ hearts, (3) pharmacological preconditioning by coronary infusion of adenosine at the measured coronary venous concentration is able to induce the same cardioprotection as conventional IPC.

### Infarct sizes and IPC effects

In both of our models IPC could reliably induce cardioprotection from the following index ischemia in wild type and CD73^-/-^ mice, resulting in a significant infarct size reduction of about 40% *in vitro* as well as *in vivo* in comparison to control groups (Figs 2 and 4). Several other groups have conducted studies to assess the relevance of extracellular adenosine formation by CD73 in IPC: In 1994 Kitakaze et al. [37] were the first to study adenosine 5’-[alpha, beta-methylene] diphosphate (AOPCP) induced inhibition of CD73^-/-^ in an open-chest model in dogs and found abolished cardioprotection, when AOPCP was administered throughout the IPC and I/R protocol. Miki et al. [38] used an in situ model of IPC and I/R in rabbits, where AOPCP induced inhibition of CD73 was unable to blunt cardioprotection. Eckle et al. [36] showed impaired IPC effects in wildtype mice with AOPCP inhibition, as well as in CD73^-/-^ mice in comparison to the wildtype. We confirm the results of Miki et al. in this study, while the differences to Eckle and Kitakaze et al. remain unclear and could only be explained by methodological differences.

We found significantly higher infarct sizes in the female CD73^-/-^ control group *in vitro* in comparison to wild type hearts (Fig 2), while there was no difference *in vivo* (Fig 4). This may be attributed to differing AAR size (global (*in vitro*) vs. regional (*in vivo*) ischemia) or also differences in heart weight, as CD73^-/-^ hearts *in vitro* tended to be larger than wild type controls (Table 1). *In vitro* experiments by other groups concerning this conundrum are not available, *in vivo* higher control infarct sizes in CD73^-/-^ mice have been reported by Eckle et al. [36], while other groups did not show diverging effects [41–43]).

There was no difference *in vivo* between male and female animals concerning the cardioprotection by IPC (Fig 4), which has been suspected in the past [44,45].

### Myocardial adenosine formation

While under normoxic conditions the rate of intracellular adenosine formation greatly exceeds the extracellular production [46], interstitial adenosine concentration rises to > 10 μM during hypoxia/ischemia [7,47] by increased release from cardiomyocytes. A hypoxia-induced inhibition of adenosine kinase [16] further potentiates the adenosine release. The amount of CD73-derived adenosine during hypoxia/ischemia in the heart is controversial, and research groups have presented differing data in different models of enzyme deletion or inhibition [20,36,37,48–50].

We used HPLC analysis to measure coronary venous nucleoside levels in the Langendorff model during the IPC trigger phase (Fig 3a), where adequate extracellular adenosine levels are most critical for receptor activation, and we measured a substantial adenosine release after IPC in WT and CD73^-/-^ hearts, without a quantitative difference in adenosine at baseline or after IPC. Inosine outflow after IPC was even higher in CD73^-/-^ hearts than in wild types, which possibly hints to an altered regulation of downstream metabolism. Our data are not in accordance with a quantitative relevance of CD73 during triggering of IPC at least in isolated saline perfused hearts, where the blood compartment cannot provide additional extracellular nucleotide substrates for adenosine formation.

### Pharmacological preconditioning

In another set of *in vitro* experiments we performed pharmacological preconditioning of wild type hearts by coronary infusion of 2.5 μM adenosine. This pharmacological preconditioning led to an infarct size reduction of 41.7% after index ischemia in comparison to control groups, which was similar to the effects of conventional IPC (Fig 3b). Pharmacological preconditioning with 10 μM intracoronary adenosine infusion has already been demonstrated to limit infarct size *in vitro* [51]. Now we additionally show that IPC-induced coronary venous adenosine levels (Fig 3a) are sufficient to reduce infarct size. Although this does not provide direct proof of the involvement of adenosine in IPC in CD73^-/-^ hearts, it nevertheless shows that the measured adenosine concentrations of WT- and CD73-deficient hearts can activate myocardial adenosine receptors and induce cardioprotection in wild type hearts.

### Left-ventricular function

LVDP and LVEDP *in vitro* showed a significant impairment after ischemia compared to baseline levels in wild type and CD73^-/-^ hearts (Table 1). IPC only slightly improved left-ventricular function during reperfusion of index ischemia compared to controls, amounting to non-significant trends in CD73-deficient and WT hearts. This was surprising at first, considering the significant myocardial salvage by IPC, but infarct size reduction without improvement of ventricular function has also been observed by other groups in the past [52,53], and mostly studies with particularly small infarct sizes have been able to show beneficial IPC effects on functional parameters [54–56]. This discrepancy of function and morphology has been partly attributed to myocardial stunning [57,58] after global ischemia, where a functional differentiation of infarcted and stunned myocardium is not possible during early phases of reperfusion, and stunning thus masks myocardial salvage by IPC. This has led others to the conclusion that cardiac function is unreliable for assessment of cardioprotection by IPC [59], which is consistent with our data.

### Limitations

Although we conclusively show that CD73 deficient mice show preserved IPC effects in two different animal models and coronary venous adenosine levels sufficient to induce cardioprotection, our data do not allow a quantitative assessment of CD73^-/-^ dependent adenosine formation. Adaptive mechanisms in knock-out animals can lead to changes in metabolic cascades, and may compensate for loss of the rate-limiting enzyme by increased activity or overexpression of other enzymes, e.g. alkaline phosphatase, which is considered to be secondary to CD73 in extracellular adenosine formation in wild types [29,60]. The CD73-knock-out used in our study was previously shown to lack induction of alkaline phosphatase or changes in plasma enzyme levels compared to wild types [61]. But since we did not study acute inhibition in wild types, we cannot exclude such compensatory effects in the CD73^-/-^ heart to be the basis for the observed preservation of cardioprotection. Additionally, recent studies hypothesize a very complex dynamic regulation of extracellular nucleotide breakdown, which may also depend on specific location in the vasculature, target organ or concentration of extracellular nucleotide levels [62].

Also, it is known that slight differences in knock-out generation may have profound influences on the resulting phenotype, e.g. in different strains of endothelial nitric oxide synthase knock-out mice [63,64], which may explain differences to Eckle et al. [36], who used a different CD73^-/-^ mouse for their experiments.

The fraction of adenosine released from the intracellular space has not been studied by blockade of equilibrative nucleoside transporters [65], which would have enabled conclusions about intra- vs extracellular adenosine formation.

On another note, we did not determine the IPC threshold by reduction of length or number of preconditioning cycles in exact measures, but understanding the threshold concept of IPC is crucial. The Downey group [7,10,66] showed that differential inhibition of main trigger substances (adenosine, bradykinin, opioids) does not completely abrogate cardioprotection by IPC, but rather increases the necessary number or duration of preconditioning I/R cycles to induce protection. Loss of CD73-derived adenosine in CD73^-/-^ may have an influence on the preconditioning threshold, but preserved intracellular adenosine formation and release of bradykinin and opioids pushed its effect below the detection limit in our setting.

## Conclusions

We analyzed effects of CD73 deficiency on IPC induction and infarct sizes in mouse hearts *in vivo* and *in vitro*, and we found no differences caused by genetic inactivation of CD73 in comparison to the wild type. Additionally, overall adenosine and inosine production in CD73^-/-^ hearts during the trigger phase of ischemic preconditioning was not reduced and the adenosine concentration released was shown to be sufficient for the induction of IPC in wild type hearts. Relevant effects of the sex could be excluded *in vivo*. Cardiac function in vitro was found to be an unrealiable parameter for assessment of cardioprotection by IPC. Our results confirm in two independent animal models, that genetic CD73^-/-^ disruption does not influence cardioprotection by early phase IPC. Further research is needed to determine the relative importance of CD73 and other key enzymes of nucleotide metabolism for extracellular ischemic adenosine formation.

## Supporting Information

S1 File Functional and metabolic parameters and infarct sizes of isolated perfused hearts. (PDF)

S2 File Infarct sizes in detail of *in vivo* hearts. (PDF)
