# Supplementary material for: Extracellular Adenosine Formation by Ecto-5’-Nucleotidase (CD73) Is No Essential Trigger for Early Phase Ischemic Preconditioning
Source: PLoS One. 2015 Aug 11;10(8):e0135086. doi: 10.1371/journal.pone.0135086 (PMC4532361; doi:10.1371/journal.pone.0135086)
Supplement: S1 File — (PDF) [file pone.0135086.s001.pdf]

S1 File: Functional and metabolic parameters and infarct sizes of isolated perfused hearts

Wild-type, no IPC	heart weight	infarct size	LVDP (basal)	LVDP (reperfusion)	LVEDP (basal)	LVEDP (reperfusion)	dP/dt max (basal)	dP/dt min (basal)	dP/dt max (reperfusion)	dP/dt min (reperfusion)	CorFlow (basal)	adenosine (basal)	adenosin (control)	inosine (basal)	inosine (control)
	mg	% AAR	mmHg	mmHg	mmHg	mmHg	mmHg/sec	mmHg/sec	mmHg/sec	mmHg/sec	ml/min/g	pmol/ml	pmol/ml	pmol/ml	pmol/ml
03.03.08 M1	150	40,4	105	61	10,3	23,5	4580	-3734	3123	-1857	26	75,3	27,9	96,3	17
06.03.08 M3	120	37	115	51	16,2	37,5	4613	-3854	2448	-1592	20	7,3	9,7	29,1	29,5
11.03.08 M1	130	54,4	142	57,6	7,2	43,1	5771	-4172	2537	-1804	23,8	9,8	18,6	45,5	21,7
14.03.08 M1	120	46,4	95	21,7	13,5	54	3757	-2856	982	-535	24,2	10,9	212	3,5	110,3
18.03.08 M1	140	42,6	76	32	11,3	21	3409	-2544	1727	-896	15,7	21,2	203,2	118,4	113,4
20.03.08 M2	100	51,1	104	43	9,5	27	4648	-3604	2355	-1450	27	56,4	56,5	59,2	126,6
27.03.08 M2	120	49,8	123	24	7,4	51	5136	-4260	1239	-830	22,5	17,4	15,9	94,8	74,9
**Mean**	**125,7**	**46,0**	**108,6**	**41,5**	**10,8**	**36,7**	**4559,1**	**-3574,9**	**2058,7**	**-1280,6**	**22,7**	**28,3**	**77,7**	**63,8**	**70,5**
**SD**	**16,2**	**6,3**	**21,0**	**15,9**	**3,2**	**13,3**	**793,6**	**646,7**	**768,3**	**522,6**	**3,9**	**26,6**	**90,0**	**41,2**	**47,5**
**n =**	**7**	**7**	**7**	**7**	**7**	**7**	**7**	**7**	**7**	**7**	**7**	**7**	**7**	**7**	**7**

Wild-type, IPC	heart weight	infarct size	LVDP (basal)	LVDP (reperfusion)	LVEDP (basal)	LVEDP (reperfusion)	dP/dt max (basal)	dP/dt min (basal)	dP/dt max (reperfusion)	dP/dt min (reperfusion)	CorFlow (basal)	adenosine (basal)	adenosine (post IPC)	inosine (basal)	inosine (post IPC)
	mg	% AAR	mmHg	mmHg	mmHg	mmHg	mmHg/sec	mmHg/sec	mmHg/sec	mmHg/sec	ml/min/g	pmol/ml	pmol/ml	pmol/ml	pmol/ml
26.02.08 M1	220	32,3	114	54,4	3	9,6	3970	-3702	1958	-1790	14,5	19	1705,3	70,2	3852,2
28.02.08 M1	110	21,3	99,3	57,3	18,6	31	4215	-3328	2948	-1742	25,5	15,2	483,8	28,9	4529,9
05.03.08 M1	140	25,6	140	59	10,6	37,9	5842	-3908	2464	-1694	15,7	5,3	1622	29,2	6605,5
10.03.08 M1	150	23,2	150	68	8,4	36,7	5963	-4357	2832	-2007	20	15	2688	31,2	8820,7
12.03.08 M2	140	33,9	116	48,7	14,5	44,4	5286	-4052	2609	-1516	18,6	13,4	2172	81,9	6719,5
17.03.08 M1	130	26,9	108	59	10,3	28	4224	-3705	2845	-2017	23,8	23,6	1807,8	93,2	4546,5
25.03.08 M1	140	24,5	96	51	10,4	19,6	3967	-3002	2394	-1549	17,9	11,4	794,7	26,9	2652,1
**Mean**	**147,1**	**26,8**	**117,6**	**56,8**	**10,8**	**29,6**	**4781,0**	**-3722,0**	**2578,6**	**-1759,3**	**19,4**	**14,7**	**1610,5**	**51,6**	**5389,5**
**SD**	**34,5**	**4,7**	**20,3**	**6,3**	**4,9**	**11,9**	**887,8**	**451,1**	**342,8**	**198,6**	**4,0**	**5,8**	**759,3**	**29,0**	**2094,7**
**n =**	**7**	**7**	**7**	**7**	**7**	**7**	**7**	**7**	**7**	**7**	**7**	**7**	**7**	**7**	**7**

KO, no IPC	heart weight	infarct size	LVDP (basal)	LVDP (reperfusion)	LVEDP (basal)	LVEDP (reperfusion)	dP/dt max (basal)	dP/dt min (basal)	dP/dt max (reperfusion)	dP/dt min (reperfusion)	CorFlow (basal)	adenosine (basal)	adenosin (control)	inosine (basal)	inosine (control)
	mg	% AAR	mmHg	mmHg	mmHg	mmHg	mmHg/sec	mmHg/sec	mmHg/sec	mmHg/sec	ml/min/g	pmol/ml	pmol/ml	pmol/ml	pmol/ml
06.03.08 M2	170	47,9	120	25,9	17,4	62,9	4853	-4003	1153	-732	16,5	6,3	21,9	68,4	47
07.03.08 M2	180	69,6	144	18	11,2	55,6	7012	-4990	812	-572	10,6	238,2	14,4	344,6	16,8
11.03.08 M2	120	54,1	156	54	3,2	32,4	6278	-4389	2197	-1730	23,3	17,6	67,1	82,1	80,8
18.03.08 M2	150	52,6	83	10,9	30	48,6	3720	-2632	1458	-905	15,3	15	128,6	92,8	41,2
19.03.08 M2	140	63	114	17,9	11,7	61,7	5137	-3907	816	-564	20,7	2,9	48,8	40,3	43,5
26.03.08 M1	150	54,8	118	34	3,1	35,6	5109	-4024	1707	-997	17,3	14	52,3	75,4	86,2
27.03.08 M1	160	50,9	106	33	8,8	39,8	4118	-3690	1583	-1066	17,5	8,4	59,7	72,2	90,3
**Mean**	**152,9**	**56,1**	**120,1**	**27,7**	**12,2**	**48,1**	**5175,3**	**-3947,9**	**1389,4**	**-938,0**	**17,3**	**43,2**	**56,1**	**110,8**	**58,0**
**SD**	**19,8**	**7,6**	**24,1**	**14,3**	**9,3**	**12,5**	**1150,0**	**717,5**	**502,2**	**400,9**	**4,0**	**86,1**	**37,3**	**104,3**	**27,9**
**n =**	**7**	**7**	**7**	**7**	**7**	**7**	**7**	**7**	**7**	**7**	**7**	**7**	**7**	**7**	**7**

KO, IPC	heart weight	infarct size	LVDP (basal)	LVDP (reperfusion)	LVEDP (basal)	LVEDP (reperfusion)	dP/dt max (basal)	dP/dt min (basal)	dP/dt max (reperfusion)	dP/dt min (reperfusion)	CorFlow (basal)	adenosine (basal)	adenosine (post IPC)	inosine (basal)	inosine (post IPC)
	mg	% AAR	mmHg	mmHg	mmHg	mmHg	mmHg/sec	mmHg/sec	mmHg/sec	mmHg/sec	ml/min/g	pmol/ml	pmol/ml	pmol/ml	pmol/ml
06.03.08 M1	150	24,3	132	51	14,4	35,9	5352	-4815	2392	-1608	20,7	15,2	1775,1	31,3	8807,8
07.03.08 M1	200	35,4	135	71	16,8	34,5	6387	-4609	2972	-2184	15,5	23	1063,9	70,9	6927,9
12.03.08 M1	150	24,2	98	51,5	15	45,3	4460	-2973	2723	-1639	12,7	10,9	2936,3	72,8	6405,6
14.03.08 M2	140	20,1	134	68	8,9	18,6	5459	-4162	2909	-2296	19,3	24,8	2642,9	98,9	15030
19.03.08 M1	170	24,9	120	23	8,1	30	6037	-4310	1116	-640	21,2	14	2477,2	80	16515,7
20.03.08 M1	160	28,5	117	42,5	10,5	41,5	5121	-4077	2264	-1334	24,4	26,9	2502,5	80,4	14968,4
25.03.08 M2	170	26,1	119	49,5	10,6	37,5	5374	-4106	2642	-1597	18,2	5,9	2667,9	32,2	13745,2
26.03.08 M2	180	21,6	123	50	8,1	39,8	5648	-4078	2516	-1480	17,8	13,6	1645,2	94,3	10430,7
**Mean**	**165,0**	**25,6**	**122,3**	**50,8**	**11,6**	**35,4**	**5479,8**	**-4141,3**	**2441,8**	**-1597,3**	**18,7**	**16,8**	**2213,9**	**70,1**	**11603,9**
**SD**	**19,3**	**4,7**	**12,1**	**14,9**	**3,4**	**8,2**	**580,5**	**544,6**	**587,5**	**511,8**	**3,6**	**7,4**	**644,0**	**25,5**	**3961,4**
**n =**	**8**	**8**	**8**	**8**	**8**	**8**	**8**	**8**	**8**	**8**	**8**	**8**	**8**	**8**	**8**

Experiments for pharmacological preconditioning

WT, no IPC	heart weight	infarct size	LVDP (basal)	LVDP (reperfusion)	LVEDP (basal)	LVEDP (reperfusion)	dP/dt max (basal)	dP/dt min (basal)	dP/dt max (reperfusion)	dP/dt min (reperfusion)	CorFlow (basal)
	mg	% AAR	mmHg	mmHg	mmHg	mmHg	mmHg/sec	mmHg/sec	mmHg/sec	mmHg/sec	ml/min/g
23.05.08 M1	170	65,8	133,56	28,29	7,96	52,08	6052,99	-4019,08	1302,42	-842,62	17,9
30.05.08 M1	150	35,9	129,67	43,19	8,9	31,77	4490,23	-3682,9	2065,02	-1377,5	18,5
19.06.08 M1	150	42,9	163,4	45,95	6,35	30,19	6241,94	-4665,2	2266,52	-1490,25	21,9
14.07.08 M1	150	59,0	121,14	30,65	5,28	37,21	5940,47	-4722,53	1604,68	-1039,46	20,9
15.07.08 M1	170	63,9	115,12	23,12	7,13	45,46	5282,85	-4313,06	1056,67	-698,14	16,6
15.07.08 M2	160	64,3	123,43	27,83	7,22	39,21	6271,67	-4485,76	1418,85	-878,57	18,7
15.07.08 M3	180	68,2	118,65	17,25	7,25	28,72	5196,89	-4628,61	843,1	-550,45	18,1
18.08.08 M1	180	66,9	121,55	24,99	2,66	46,37	4956,98	-3510,23	1056,48	-762,97	14,3
**Mean**	**163,8**	**58,3**	**128,3**	**30,2**	**6,6**	**38,9**	**5554,3**	**-4253,4**	**1451,7**	**-955,0**	**18,4**
**SD**	**13,0**	**12,2**	**15,3**	**9,8**	**1,9**	**8,5**	**662,9**	**466,1**	**502,8**	**328,9**	**2,4**
**n =**	**8**	**8**	**8**	**8**	**8**	**8**	**8**	**8**	**8**	**8**	**8**

WT, IPC	heart weight	infarct size	LVDP (basal)	LVDP (reperfusion)	LVEDP (basal)	LVEDP (reperfusion)	dP/dt max (basal)	dP/dt min (basal)	dP/dt max (reperfusion)	dP/dt min (reperfusion)	CorFlow (basal)
	mg	% AAR	mmHg	mmHg	mmHg	mmHg	mmHg/sec	mmHg/sec	mmHg/sec	mmHg/sec	ml/min/g
27.05.08 M1	150	26,3	136,09	61,93	8,19	24,73	5550,62	-3895,09	2560,05	-1951,13	19,1
11.07.08 M2	170	53,1	141,08	31,36	2,32	24,72	5173,4	-4542,97	1131,46	-956,39	22,4
18.08.08 M2	170	37,5	115,08	42,86	10,52	41,5	4677,84	-3389,02	1837,09	-1293,75	15,9
19.08.08 M1	180	34,3	109,34	38,78	9,14	38,85	5004,37	-3708,94	1997,53	-1232,97	17,8
19.08.08 M2	200	36,7	129,75	50,66	10,11	39,74	6289,3	-4815,85	2673,99	-1667,56	18,1
**Mean**	**174,0**	**37,6**	**126,3**	**45,1**	**8,1**	**33,9**	**5339,1**	**-4070,4**	**2040,0**	**-1420,4**	**18,7**
**SD**	**18,2**	**9,7**	**13,6**	**11,7**	**3,3**	**8,4**	**617,3**	**592,6**	**620,6**	**390,3**	**2,4**
**n =**	**5**	**5**	**5**	**5**	**5**	**5**	**5**	**5**	**5**	**5**	**5**

WT, + adenosine	heart weight	infarct size	LVDP (basal)	LVDP (reperfusion)	LVEDP (basal)	LVEDP (reperfusion)	dP/dt max (basal)	dP/dt min (basal)	dP/dt max (reperfusion)	dP/dt min (reperfusion)	CorFlow (basal)
	mg	% AAR	mmHg	mmHg	mmHg	mmHg	mmHg/sec	mmHg/sec	mmHg/sec	mmHg/sec	ml/min/g
07.05.08 M1	120	27,3	113,03	49,89	11,55	33,93	5478,07	-3389,44	2400,53	-1624,54	24,5
25.05.08 M1	170	27,1	120,07	62,26	13,68	22,28	3936,91	-3672,1	2224,88	-1913,4	18,6
11.07.08 M1	180	39,8	146,91	49,67	14,89	24,64	5229,04	-3775,54	1856,1	-1654,16	16,2
20.08.08 M1	150	36,1	128,67	71,25	16,53	19,88	6295,38	-4955,11	3652,92	-2457,95	20,1
22.08.08 M1	150	35,5	103,17	53,41	12,05	28,27	4016,25	-3717,57	2597,32	-1743,29	16,4
22.08.08 M2	140	38,3	121,3	36,6	15,44	29,25	4916,23	-4119,92	1896,4	-1245,57	24,5
**Mean**	**151,7**	**34,0**	**122,2**	**53,8**	**14,0**	**26,4**	**4978,6**	**-3938,3**	**2438,0**	**-1773,2**	**20,1**
**SD**	**21,4**	**5,5**	**14,9**	**11,9**	**2,0**	**5,1**	**901,2**	**550,3**	**660,3**	**401,0**	**3,7**
**n =**	**6**	**6**	**6**	**6**	**6**	**6**	**6**	**6**	**6**	**6**	**6**
