# Supplementary material for: Extracellular Adenosine Formation by Ecto-5’-Nucleotidase (CD73) Is No Essential Trigger for Early Phase Ischemic Preconditioning
Source: PLoS One. 2015 Aug 11;10(8):e0135086. doi: 10.1371/journal.pone.0135086 (PMC4532361; doi:10.1371/journal.pone.0135086)
Supplement: S2 File — (PDF) [file pone.0135086.s002.pdf]

S2 File: Infarct sizes in detail of *in vivo* hearts

Groups overall

**Wild-type, no IPC**	**body weight**	**Infarct Size**
	g	%AAR
2009_07_29_45_90_I_m	26,6	44,8
2009_07_30_45_90_II_m	24,9	47,3
2009_08_03_45_90_I_m	26,4	38,2
2009_08_04_45_90_I_f	26,7	47,5
2009_08_04_45_90_III_m	27,5	22,5
2009_08_07_45_90_II_f	22,7	46,0
2009_08_10_45_90_II_m	28,4	47,7
2009_09_22_45_90_I_m	24,4	51,5
2009_09_26_45_90_II_m		59,6
2009_09_30_45_90_I_f	23,0	41,2
2009_10_12_45_90_I_f	21,2	46,8
2009_10_14_45_90_I_f	23,7	50,1
**Mean**	**25,0**	**45,3**
**SD**	**2,3**	**8,9**
**n=**	11,0	12,0

**KO, no IPC**	**body weight**	**Infarct Size**
	g	%AAR
2009_08_05_45_90_II_m_KO	35,5	45,4
2009_08_08_45_90_I_m_KO	29,8	44,3
2009_08_11_45_90_I_m_KO	28,3	44,5
2009_09_24_45_90_I_m_KO	28,1	43,3
2009_09_27_45_90_I_m_KO	25,6	33,3
2009_09_29_45_90_I_m_KO	29,5	53,7
2009_10_13_45_90_II_f_KO	23,5	32,8
2009_10_14_45_90_II_f_KO	21,5	34,9
2009_10_15_45_90_I_f_KO	22,8	40,2
2009_10_24_45_90_I_f_KO	21,1	51,3
2009_10_25_45_90_I_f_KO	22,5	25,8
2009_10_31_45_90_I_f_KO	24,5	34,1
2009_11_01_45_90_I_f_KO	23,0	43,0
**Mean**	**25,8**	**40,5**
**SD**	**4,2**	**8,0**
**n=**	13,0	13,0

**Wild-type, IPC**	**body weight**	**Infarct Size**
	g	%AAR
2009_07_30_45_90_I_m_IPC	24,6	25,2
2009_07_31_45_90_I_m_IPC	26,3	22,0
2009_08_03_45_90_II_m_IPC	24,8	15,4
2009_08_04_45_90_II_f_IPC	29,5	32,3
2009_08_07_45_90_I_f_IPC	20,7	28,8
2009_08_10_45_90_I_m_IPC	26,4	12,8
2009_09_22_45_90_II_f_IPC	22,0	24,1
2009_09_23_45_90_I_m_IPC	22,3	40,0
2009_09_23_45_90_II_m_IPC	20,8	36,2
2009_09_26_45_90_I_m_IPC	28,2	23,4
2009_10_15_45_90_II_f_IPC	24,2	31,3
2009_10_19_45_90_I_f_IPC	23,9	24,5
**Mean**	**24,5**	**26,3**
**SD**	**2,8**	**7,9**
**n=**	12,0	12,0
**IS Reduktion (%)**		**41,8**

**KO, IPC**	**body weight**	**Infarct Size**
	g	%AAR
2009_08_05_45_90_I_m_KO_IPC	36,0	11,5
2009_08_11_45_90_II_m_KO_IPC	30,8	20,6
2009_08_12_45_90_I_m_KO_IPC	30,9	32,5
2009_09_24_45_90_II_m_KO_IPC	27,3	27,7
2009_09_27_45_90_II_m_KO_IPC	25,8	16,1
2009_09_29_45_90_II_m_KO_IPC	25,6	26,3
2009_10_13_45_90_I_f_KO_IPC	21,7	13,0
2009_10_16_45_90_I_f_KO_IPC	20,9	30,3
2009_10_16_45_90_II_f_KO_IPC	22,4	27,4
2009_10_24_45_90_II_f_KO_IPC	20,3	21,5
2009_10_30_45_90_I_f_KO_IPC	21,9	16,9
2009_10_30_45_90_II_f_KO_IPC	22,6	24,1
2009_11_03_45_90_I_f_KO_IPC	24,9	26,1
**Mean**	**25,5**	**22,6**
**SD**	**4,7**	**6,6**
**n=**	13,0	13,0
**IS Reduktion (%)**		**44,2**

Grouped by sex

Wild-type, male, no IPC	body weight	Infarct Size
	g	%AAR
2009_07_29_45_90_I_m	26,6	44,8
2009_07_30_45_90_II_m	24,9	47,3
2009_08_03_45_90_I_m	26,4	38,2
2009_08_04_45_90_III_m	27,5	22,5
2009_08_10_45_90_II_m	28,4	47,7
2009_09_22_45_90_I_m	24,4	51,5
2009_09_26_45_90_II_m		59,6
**Mean**	**26,4**	**44,5**
**SD**	**1,5**	**11,7**
**n=**	6,0	7,0

Wild-type, female, no IPC	body weight	Infarct Size
	g	%AAR
2009_08_04_45_90_I_f	26,7	47,5
2009_08_07_45_90_II_f	22,7	46,0
2009_09_30_45_90_I_f	23,0	41,2
2009_10_12_45_90_I_f	21,2	46,8
2009_10_14_45_90_I_f	23,7	50,1
**Mean**	**23,5**	**46,3**
**SD**	**2,0**	**3,2**
**n=**	5,0	5,0

Wild-type, male, IPC	body weight	Infarct Size
	g	%AAR
2009_07_30_45_90_I_m_IPC	24,6	25,2
2009_07_31_45_90_I_m_IPC	26,3	22,0
2009_08_03_45_90_II_m_IPC	24,8	15,4
2009_08_10_45_90_I_m_IPC	26,4	12,8
2009_09_23_45_90_I_m_IPC	22,3	40,0
2009_09_23_45_90_II_m_IPC	20,8	36,2
2009_09_26_45_90_I_m_IPC	28,2	23,4
**Mean**	**24,8**	**25,0**
**SD**	**2,5**	**10,0**
**n=**	7,0	7,0
**IS Reduktion (%)**		**43,8**

Wild-type, female, IPC	body weight	Infarct Size
	g	%AAR
2009_08_04_45_90_II_f_IPC	29,5	32,3
2009_08_07_45_90_I_f_IPC	20,7	28,8
2009_09_22_45_90_II_f_IPC	22,0	24,1
2009_10_15_45_90_II_f_IPC	24,2	31,3
2009_10_19_45_90_I_f_IPC	23,9	24,5
**Mean**	**24,1**	**28,2**
**SD**	**3,4**	**3,8**
**n=**	5,0	5,0
**IS Reduktion (%)**		**39,1**

KO, male, no IPC	body weight	Infarct Size
	g	%AAR
2009_08_05_45_90_II_m_KO	35,5	45,4
2009_08_08_45_90_I_m_KO	29,8	44,3
2009_08_11_45_90_I_m_KO	28,3	44,5
2009_09_24_45_90_I_m_KO	28,1	43,3
2009_09_27_45_90_I_m_KO	25,6	33,3
2009_09_29_45_90_I_m_KO	29,5	53,7
**Mean**	**29,5**	**44,1**
**SD**	**3,3**	**6,5**
**n=**	6,0	6,0

KO, female, no IPC	body weight	Infarct Size
	g	%AAR
2009_10_13_45_90_II_f_KO	23,5	32,8
2009_10_14_45_90_II_f_KO	21,5	34,9
2009_10_15_45_90_I_f_KO	22,8	40,2
2009_10_24_45_90_I_f_KO	21,1	51,3
2009_10_25_45_90_I_f_KO	22,5	25,8
2009_10_31_45_90_I_f_KO	24,5	34,1
2009_11_01_45_90_I_f_KO	23,0	43,0
**Mean**	**22,7**	**37,4**
**SD**	**1,2**	**8,2**
**n=**	7,0	7,0

KO, male, IPC	body weight	Infarct Size
	g	%AAR
2009_08_05_45_90_I_m_KO_IPC	36,0	11,5
2009_08_11_45_90_II_m_KO_IPC	30,8	20,6
2009_08_12_45_90_I_m_KO_IPC	30,9	32,5
2009_09_24_45_90_II_m_KO_IPC	27,3	27,7
2009_09_27_45_90_II_m_KO_IPC	25,8	16,1
2009_09_29_45_90_II_m_KO_IPC	25,6	26,3
**Mean**	**29,4**	**22,4**
**SD**	**4,0**	**7,8**
**n=**	6,0	6,0
**IS Reduktion (%)**		**49,1**

KO, female, IPC	body weight	Infarct Size
	g	%AAR
2009_10_13_45_90_I_f_KO_IPC	21,7	13,0
2009_10_16_45_90_I_f_KO_IPC	20,9	30,3
2009_10_16_45_90_II_f_KO_IPC	22,4	27,4
2009_10_24_45_90_II_f_KO_IPC	20,3	21,5
2009_10_30_45_90_I_f_KO_IPC	21,9	16,9
2009_10_30_45_90_II_f_KO_IPC	22,6	24,1
2009_11_03_45_90_I_f_KO_IPC	24,9	26,1
**Mean**	**22,1**	**22,8**
**SD**	**1,5**	**6,1**
**n=**	7,0	7,0
**IS Reduktion (%)**		**39,2**
